# Management of Clinically Insignificant Residual Fragments following Shock Wave Lithotripsy

**DOI:** 10.1155/2012/320104

**Published:** 2012-05-31

**Authors:** Elisa Cicerello, Franco Merlo, Luigi Maccatrozzo

**Affiliations:** Unità Complessa di Urologia, Ospedale Regionale Ca'Foncello, Treviso, Italy

## Abstract

Clinically insignificant residual fragments (CIRFs) are small fragments (less than 5 mm) that are present in upper urinary tract at the time of regular post-SWL followup. The term is controversial because they may remain silent and asymptomatic or become a risk factor for stone growth and recurrence, leading to symptomatic events, and need further urologic treatment. Although a stone-free state is the desired outcome of surgical treatment of urolithiasis, the authors believe that the presence of noninfected, nonobstructive, asymptomatic residual fragments can be managed metabolically in order to prevent stone growth and recurrence. Further urologic intervention is warranted if clinical indications for stone removal are present.

## 1. Introduction

With the introduction of shock wave lithotripsy (SWL) in 1980, the treatment of renal calculi was revolutionised. Few medical innovations have had the dramatic effect of SWL which quickly became the treatment of choice for most upper-tract calculi [1]. However, as our approach to the treatment of urinary calculi has changed, so has our concept of what constitutes successful treatment. When open surgery was the standard treatment for the management of renal calculi, the presence of residual stones suggested a failed procedure, even if those remaining stones were small [2]. SWL does not remove stones; it disintegrates them producing fragments which must be passively excreted. However, the clearance of the fragments produced by shock waves is not immediate since as many as 85% of patients have radiological evidence of residual fragments when discharged from hospital [3]. The residual fragments are defined as all fragments remaining in the kidney 3 months after the last session of SWL. Among these fragments, those larger than 5 mm are generally considered as failures of the SWL session. The residual fragments with diameter less than 5 mm that are asymptomatic and noninfected are expected to pass spontaneously without further treatment, leading to the definition of clinically insignificant residual fragments (CIRFs) [4]. In case of persistence within the upper urinary tract, these fragments may grow and gain clinical relevance again, becoming symptomatic or requiring intervention [5].

This article paper the implications of residual fragments after lithotripsy and suggests guidelines for their management.

## 2. Incidence of Residual Fragments

 Residual fragments are common after SWL. At the discharge, fragments less than 5 mm have been described in 85% to 96% of patients with calcium [3, 6] and in 92% with infected stones [7]. The majority of these fragments will be passed within a few weeks. With increasing renal persistence of residual fragments, the probability of stone clearance seems to decrease [8].

In the absence of symptoms, most stone centers recommended radiographic evaluation approximately 1 month after SWL and at varying intervals thereafter as deemed clinically necessary. With such followup, studies of large numbers of patients reveal that 24% to 36% will have residual fragments up to 3 months after SWL [3, 6, 9, 10].

Clinical experience has been able to identify several prognostic factors that decrease the incidence of residual fragments after SWL. Stone size (diameter more than 20 mm), multiple stones, and stone composed primarily of cystine, brushite, or calcium oxalate monohydrate are less likely to be cleared completely after SWL and more likely associated with residual fragments [11–14]. When congenital renal anomalies (e.g., horseshoe, malrotated and duplex kidneys) or distorted urinary tract are present, SWL could be the treatment of choice when the stone size is less than 20 mm. Anterograde or retrograde ancillary procedures or multiple SWL sessions could be required with consequent morbidity and highest cost-efficiency, while anatomic anomalies do not appear to have a significant impact on stone-free rate if percutaneous lithotripsy or more frequently ureteroscopy is performed [15–20]. In the case of transplanted kidneys, SWL could be successfully performed only for small stones treating the patient in prone position with ultrasound targeting. [21, 22]. The clearance of fragments is, moreover, delayed for lower pole renal calculi and certain spatial anatomic factors, such as the infundibulopelvic angle, infundibular length and width [17, 23, 24]. However, another study has reported that the clearance of lower calyx stones could not be influenced by collecting system anatomy [25]. Morbid obesity, independent of previous factors, also impacts on stone-clearance rates [14].

Taking these studies into account, evaluation of patients prior to SWL is important, and the use of imaging in the decision process can help to identify suitable patients for shock wave treatment. More recently, nomograms and artificial neural networks have been created in predicting the outcome with the use of computed tomography attenuation values and skin-to-stone distance. In addition, modifications in shock wave delivery by altering shock wave rate and voltage and the use of expulsive and chemolytic treatment have been researched in an effort to improve shock wave efficacy [14, 26].

## 3. Diagnosis of Residual Fragments

While the presence of residual fragments is usually reported as a function of the findings on plain abdominal radiographs, plain films have significant limitations.

The initial report that compared endoscopic and radiological evaluation of residual fragments rates following percutaneous nephrostolithotomy and SWL demonstrated that plain abdominal radiographs and renal tomography overestimated stone-free rates by 35% and 17%, respectively, compared with flexible nephroscopy [27]. Nephroscopy, however, is routinely feasible postoperatively only in patients who have undergone a percutaneous nephrostolithotomy and still have a nephrostomy tube in place.

Given the limitations of plain films, the true incidence of residual stones is therefore probably higher than generally reported. These facts result from superimposition of bowel gas, feces, and soft-tissue calcifications. As such, at least, some of the reported post-SWL recurrences represent growth of missed or nonradiologically visible residual fragments. Some reports have suggested that tomograms detect more stones than the original reported by plain films. Jewett et al. studied interobserver and intraobserver reduced variability in assessing the presence of post-ESWL residual fragments reading plain films and tomography together [28].

Several reports have addressed the sensitivity of ultrasound in the detection of urolithiasis to be between 65% and 95% [29, 30]. The advantage of ultrasound relies on the fact that all stones, independent of their composition, present an acoustic-impedance mismatch with the surrounding tissues and attenuate sound to a greater degree. However, ultrasound is inadequate in quantifying the stone burden and generally does not permit differentiation on intact stones from fragmented stones.

Nowdays, the most accurate diagnostic test to address the presence of residual fragments is noncontrast spiral CT that have proved superior to plain radiography, linear tomography and ultrasound in detecting postprocedural residual fragments [31–34]. Spiral CT also was shown to have superior sensitivity and equal specificity to intravenous urography in detecting renal stones and demonstrates equal sensitivity in the detection of ureteral dilation and can detect stone of various composition. Therefore, it has been reported that selective use of flexible nephroscopy after percutaneous nephrostolithotomy based on positive CT finding will avoid an unnecessary operation. Nephroscopy or flexible ureteroscopy could be necessary if second look for removal of residual fragments has been planned [35].

## 4. Fate of Residual Stones

Residual fragments may be important for a variety of reasons: they may act as a nidus for recurrent stone growth, they can become acutely dislodged and cause significant obstruction with pain and infection, or they may be the source of persistent infection.

After stone fragmentation, the underlying metabolic abnormalities for recurrent stone formation persist. Supersaturation of the urine with stone-forming salts or lack of stone inhibitors may accelerate the growth of residual fragments and promote new stone recurrence. Furthermore, it has been reported that in the case of patients rendered stone-free after SWL, stone-recurrence rate is higher than a similar group treated by percutaneous nephrolithotomy, without ultrasonic fragmentation [36]. Higher recurrences may be caused by microscopic sand particles migrating to dependent calyces and acting as nidus for new stone formation. By dramatically increasing the surface area of the original stone, SWL may favour new stone growth through heterogeneous nucleation and crystal aggregation by exposing the stone area to a lithogenic environment.

Several groups have reported stone recurrence from such fragments in varying percentages [4, 5, 8, 37–40] (Table 1). Bucholz et al. [40] reported a very low (2%) regrowth rate of residual fragments at a mean followup of 2.5 years, while a far higher 59% regrowth rate at a followup of 15 months was published by Khaitan et al. [38].

A prospective study followed 160 patients with 4 mm or smaller asymptomatic calcium oxalate or calcium phosphate stone fragments after SWL for a mean of 23 months. Overall the fragment regrowth rate was 18.1%, while 41.9% of fragments remained unchanged in size during long-term followup. After 5 years, 36% of fragments had passed, and the majority of these did so within the first year after SWL. Using Kaplan-Mayer estimates, the probability of fragments passing, decreasing in number or remaining stable, was 80% over 5 years. At followup, however, 43.1% of these patients had developed significant symptomatic episodes that needed intervention when stone migrated to the ureter or increased in size [37].

Historically, infection stones have required aggressive extirpative management. The urea splitting bacteria necessary for the formation of these stones may persist in the residual fragments, thus promoting a cycle of persistent infection and accelerated growth [41].

Sterilisation of residual infection stones is the goal standard of the treatment. While the role of long-term urease inhibitors in this setting is unproven, we have observed at 12 months of followup the effectiveness of prophylactic antibiotics not only on the clearance, but also on the growth and aggregation of residual infection fragments after SWL [42].

## 5. Management of Residual Stones

The management of residual fragments is controversial, especially given the potential for these fragments to grow and become clinically significant.

With the goal of improving stone-free rate, several authors have advocated early retreatment. Kring et al. retreated patients who showed residual fragments 2 months after SWL, and 60% of these patients were subsequently rendered stone-free [43]. Moon and Kim applied an additional session of SWL to small (3-4 mm) residual fragments present 1 month after SWL and achieved a 92% stone-free rate at 6 months [44]. However, although the complication rate is minimal, patients are inconvenienced, and work days are lost.

More recently, Albanis et al. evaluated the efficacy and safety of forced hydration and diuresis with limited inversion during shock wave lithotripsy in improving stone-free rate for lower pole stone clearance. Followup at 3 months showed that 83.3% of the patients belonging to the study group were rendered stone-free, whereas 71.5% were stone-free in the control group, without statically significant difference [45].

Nowadays, the cost of medical procedures must be considered. Secondary treatment could be considered in symptomatic, obstructive residual stones or in stone-associated urinary tract infection. Treatment could also be considered for asymptomatic residual stones in patients who cannot risk an episode of kidney colic (e.g., airplane pilots) or urinary tract infection (e.g., transplant patients). In these cases, ureteroscopy could be considered [19, 46].

In patients with nonobstructive, noninfected, asymptomatic residual fragments, one might consider aggressive medical therapy with the correction of underlying metabolic disorders to prevent stone growth or formation of new calculi. One or more metabolic abnormalities can be identified in up to 77% of stone-forming patients [47].

Several studies endorse the role of medical treatment after SWL, especially when residual fragments are present. Fine et al. evaluated 80 patients who underwent a full metabolic evaluation after SWL and were given selective medical therapy [48]. Specific attention was paid to the significance of residual stone fragments and their effect on stone growth or recurrent stone formation during long-term followup. In patients with residual fragments after SWL, specific medical therapy produced an 81% stone-free rate. More than half of the patients with residual stone fragments who were not managed with medical therapy showed significant stone growth at followup. Only 16% of the same group of patients on medical treatment demonstrated increase in stone size.

Our randomized prospective trial studied the effect of citrate therapy or conservative measures (dietary limitation of dairy products and salty foods and increased fluids) on residual fragments 6 to 8 weeks after SWL [42]. At a followup of 12 months, there was 75% reduction in the clearance of calcium stone fragments in patients receiving citrate treatment. Only 32% of the patients following conservative measures, however, had clearance of their calcium stone fragments. Similarly, in patients with residual-infected fragments, nonselective medical therapy with citrate cleared residual fragments in 86% of the patients, whereas only 40% of the patients following conservative measures had clearance of their infected fragments.

Another study evaluated the effect of potassium citrate on residual stone fragments after SWL for lower pole calcium oxalate urolithiasis [49]. Four weeks after SWL, 34 patients who had residual stones were randomly assigned to a citrate therapy or control group (high fluid intake recommended to achieve a minimum daily urine output of 2 L and avoidance of dietary excesses). At the 12 month followup, 44.4% of the treated patients were stone-free, whereas clearance was obtained in only 12.5% of the control group.

A randomized study confirms the effectiveness of potassium citrate in children with residual fragments 4 weeks after SWL [50]. Specific attention as paid to the significance of residual stone fragments and their effect on stone growth or recurrence in the treated and control groups (no specific preventive measure).

At 24.4 months of followup, children who had received citrate treatment showed acceptable regrowth and recurrence rates (18.1%), whereas both recurrence and regrowth were evident in most children of the control group (72.7%).

Recently, another randomized study has confirmed the preventive effects of alkaline citrate on stone recurrence as well as stone growth after SWL or percutaneous nephrolithotomy in 76 patients with calcium-containing stones [51]. At 12 months of followup, of the residual stone group, 30.8% and 9.1% of the treated and control groups were stone-free, respectively. Furthermore, increased stone size was found in 7.7% and 54.5% of the treated and control groups, respectively.

Taking these studies into account, residual fragments pose a significant risk for stone or recurrent stone formation. Medical therapy, specifically alkaline citrate, reduces the rate of stone recurrence or stone growth in patients undergoing SWL for renal calculi. Consequently, adjuvant medical therapy after SWL may actually improve stone-free rates by encouraging fragment clearance. If this is indeed the case, the administration of potassium citrate immediately after or before SWL may prove to be efficacious. Interestingly, potassium citrate appeared to be effective in a variety of metabolic anomalies, although stratification of outcomes by urinary biochemical abnormality was not performed in some studies [52]. With further study, short- or long-term potassium citrate treatment of patients undergoing SWL may prove beneficial.

## Figures and Tables

**Table 1:** Rate of regrowth of residual fragments.

Study (year)	Followup (mos)	Growth (%)
Streem et al., 1996 [37]	89	18.1
Zanetti et al., 1997 [39]	24	17
Bucholz et al., 1997 [40]	30	2.1
Candau et al., 2000 [4]	40	37
Khaitan et al., 2002 [38]	15	59
Osman et al., 2005 [8]	60	21.4
El-Nahas et al., 2006 [5]	31	13.6
